# Historic p87 Is Diagnostic for Lung Cancer Preceding Clinical Presentation by at Least 4 Years

**DOI:** 10.3390/cancers17060952

**Published:** 2025-03-12

**Authors:** Martin Tobi, Daniel Ezekwudo, Yosef Y. Tobi, Xiaoqing Zhao, Fadi Antaki, MaryAnn Rambus, Edi Levi, Harvinder Talwar, Benita McVicker

**Affiliations:** 1 John D. Dingell VA Medical Center, Detroit, MI 48201, USA; martin.tobi@va.gov (M.T.); ytobi@student.nymc.edu (Y.Y.T.); 2 Corewellhealth East William Beaumont Hospital, Royal Oak, MI 48073, USA; 3 Philadelphia VA Medical Center, Philadelphia, PA 19104, USA; xqzhao1010@gmail.com; 4 Veterans Health Administration, Durham, NC 27705, USA; fadi.antaki@va.gov; 5 Detroit VAMC, Detroit, MI 48201, USA; maryrambus639@gmail.com; 6 VA Medical Center, Wayne State University, Detroit, MI 48201, USA; edi.levi@va.gov; 7 Department of Medicine, Wayne State University, Detroit, MI 48201, USA; htalwar@med.wayne.edu; 8 Department of Internal Medicine, University of Nebraska at Omaha, Omaha, NE 68005, USA; bmcvicker@unmc.edu

**Keywords:** lung cancer, early diagnosis, Historic p87, Adnab-9, NSCLC

## Abstract

Lung cancer remains the most common cancer worldwide, with a limited prognosis despite personalized treatment regimens. Low-dose computed tomography (CT) scanning as a means of early diagnosis has been disappointing due to the high false positive rate. Other non-invasive means of testing need to be developed that offer both timely diagnosis and predict prognosis. **Methods**: In the course of stool testing in large-scale testing of 2922 patients at increased risk of CRC, we were able to ascertain 112 patients documented to have prospectively been diagnosed with lung cancer. Stool and colonic effluents were tested for p87 with anti-adenoma antibody (Adnab-9) reactivity by ELISA and Western blot. Survival data were obtained where available. **Results**: Of 112 cancers, approximately 27.6% were squamous (SSC), 17.9% were adenocarcinoma, 8% were small, 6.25% were large cell, 3.57% were designated non-small cell cancer (NSCLC), 0.89% were indeterminate, 0.89% were lepidic spread, 3.57% had metastasis, and in 31.25%, data were unavailable. In total, 49.1% of the lung cancer patients had fecal Adnab-9 testing. Overall, 60% had positive testing compared to 38%, which was significant (OR2.19 [1.06–4.53]; *p* = 0.045). Cancers with higher lethality were less likely to test positive (approximately 8.5% each for both small and large cell lung cancers) and higher, with 56% for SCC and 25% for adenocarcinoma (0% NSCLC). In the larger groups, overall survival was worse in those testing positive: 474 testing positives versus 844 days in SCC and 54 testing positive versus 749 days in adenocarcinoma patients. Most importantly, the time from a positive test to the clinical diagnosis ranged from 2.72 years for small cell, 3.13 for adenocarcinoma, 5.07 for NSCLC, 6.07 for SSC, and 6.24 for large cell cancer. In excluded cases where cancer in the lung was believed to be metastatic, 83.3% of cancers were positive. **Conclusions**: At a projected real-world sensitivity of 0.60 and specificity of 0.60, and the ability to predate diagnosis by up to 4.7 years overall, this test could help direct lung cancer screening. In addition, the Adnab-9 testing selectively detects worse tumor types (87.5%) and those with worse prognoses amongst the more common, favorable phenotypes, thus making early diagnosis possible in those patients who stand to benefit most from this strategy. Metastatic lung cancer, also detected by the test, should be identified by the follow-up imaging studies and, therefore, would not be considered to be a major pitfall.

## Simple Summary

Lung cancer remains a leading cause of cancer mortality. Early detection could decrease mortality and increase diagnosis of lung cancer at early stage. We aim in this study to discuss noninvasive approach to early detection. At a projected real-world sensitivity of 0.60 and specificity of 0.60, and the ability to predate diagnosis by up to 4.7 years overall, this test could help direct lung cancer screening. To the best of our knowledge, this is the first time such assay has been utilized in early diagnosis of lung cancer.

## 1. Introduction

Lung cancer is the most common cancer worldwide in men and women, and incidence is increasing in developing countries. Early diagnosis with reduced mortality is achievable with low-dose CT scans, but false positives and morbidity of follow-up studies threaten to disrupt screening efforts. Biomarkers to select patients at the highest risk are available, but enthusiasm is lacking due to economic considerations, and prospective studies are limited. In addition, despite gains in reduced mortality using novel small molecular weight inhibitors, it still remains, for most victims, a deadly disease. We describe prospective stool and effluent testing in a cohort of urban patients using a monoclonal antibody Adnab-9 that recognizes p87 as a constituent of Paneth cells, an effector of the innate immune system, and staining in an unrelated cohort of familial lung cancer patients and control lung tissues from the same geographic location [1]. Recently, we have used blood Ferritin level with p87 recognized by the Adnab-9 monoclonal that recognizes p87 as the denominator for the resultant ratio, which we have termed “FERAD”. We believe that the FERAD ratio is an accurate measure of the innate immune system (InImS) activity, particularly in responding to cancer and viral infections [2]. We have also used this ratio to contrast InImS in lung cancer with its frequent concomitant [3], chronic obstructive pulmonary disease (COPD).

Furthermore, previously described blood-based biomarkers for lung cancer are gaining clinical validation [4,5,6].

## 2. Methods

**Scheme 1:**
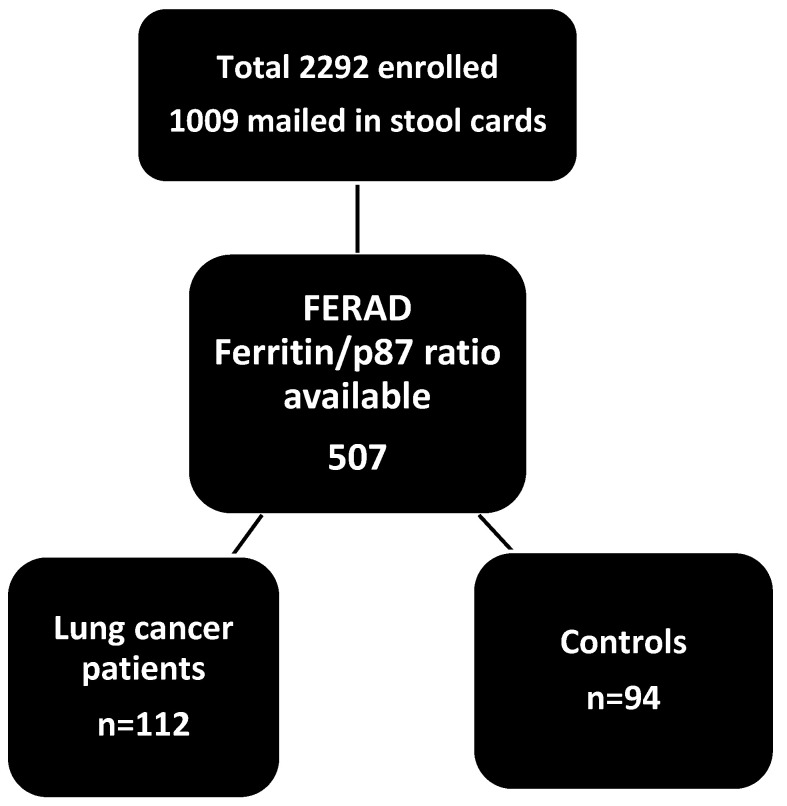
A schematic of the overall study.

Prospectively, 2922 American patients in Metropolitan Detroit were asked to submit stool samples in a CRC prospective screening study from 1996 to 2013. We used a stool ELISA and Western blotting, as described previously [7], to detect p87, a product of the innate immune system, to prospectively detect lung cancer in a cohort of patients at high risk for colorectal cancer. Controls were selected on the basis of criteria of no diagnosed lung cancer, other cancer, or family history of cancer. Other criteria used were non-smokers, no alcohol intake, and non-diabetic patients. Patients and controls were approximately 50–80 years old, and the male-to-female ratios were similar (5:1 and 10:1). Overall, 67% of patients and 45 % of controls were African American. Immunohistochemical (IHC) staining samples of lung tissues of 28 patients with lung cancer from patients seen at the Karmanos Cancer Institute were collected retrospectively, and the IHC staining was performed as described previously [8]. Commercially available lung tissue arrays with malignant and benign tissues were stained with anti-p87 monoclonal Adnab-9, and ELISA of tissue extracts from 3 bronchoalveolar (lepidic spread) cell lines (a kind gift of Dr. Galli Hilman) was performed. A schematic of the overall study is shown below (Scheme 1).

Results: 112 Lung cancers were detected on follow-up, 8.25 ± 5.63 years before the clinical diagnosis. These data were available in 52 patients (46%). Only 44% of the entire population submitted a stool specimen and reflected accordingly, while 46% of patients with lung cancer did not provide a stool sample. The sensitivity was 60% vs. 38% in 44 controls, and the difference was significant (OR2.19 [1.06–4.53]; *p* = 0.045). In the group who submitted stool samples, twenty were adenocarcinomas; thirty-one were squamous cell cancers; nine were small cell; seven were large cell; four were non-small cell; four were metastatic; one was indeterminate; and one had lepidic spread features; data were unavailable in 35 patients (29%). Patients are summarized in Table 1.

We found that about half the patients ultimately diagnosed with lung cancer had either stool ELISA or Western blot positivity for Adnab-9 compared to 14 patients that were alive, and 48 had expired (22.6% survival). Seventy-four were black, thirty-seven were white, and one was unknown. One hundred and one were male, and nine were female (two unknown); the average age was 63.78 ± 10.82 years at the time of enrollment. In total, 49 were actively smoking, and 26 were not. Quantitively, data were available in over half the patients: six had quit, twenty-four had <30 pyh, fourteen < 60 pyh, twelve had < 100 pyh, and fifteen had > 100 pyh. Overall, 58 had a BMI < 28 kg/m, and 24 were greater where available. Twenty-one did not have a second cancer diagnosis. According to tumor type, adenocarcinoma lead-in time from test to diagnosis was 10.96 ± 6.41 years (age 57.77 ± 9.43); 4.09 ± 3.39 years (age 65.20 ± 8.08) for large cell; 4.55 ± 4.20 years (age 65.00 ± 7.53) for non-small cell cancer; 8.38 ± 6.53 years (age 64.15 ± 11.72) for squamous cell cancer; 3.31 ± 3.00 years (age 72.33 ± 7.79) for small cell cancer; and 9.46± 5.34 years was the lead-in time where the type of cancer was unknown (age 65.93 ± 10.4).

**Figure 1:**
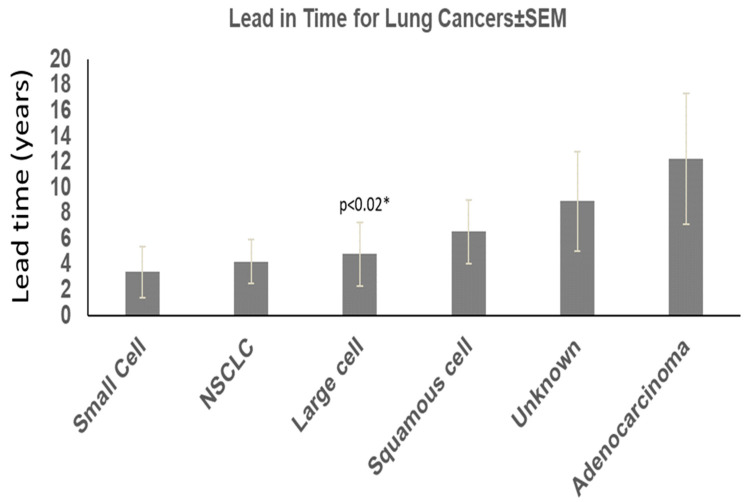
Lead-in time and lung cancer types.

The “*” (Figure 1) shows the significance when comparing lead-in time for adenocarcinoma versus large cell cancers. The other significant differences between cancer cases and controls were as follows: age where patients with cancer over 50 years of age predominated over control cases (78% versus 62%; 2.16 (1.37–3.40) chi-square with Yates correction; Black African ancestry (67 vs. 52%; 1.83 [1.22–2.75]; *p* < 0.005; less obesity (BMI < 80 m/kg^2^ in the lung cancer group (29 vs. 51%; 0.39 [0.24–0.64]; *p* < 0.0002); smoking 65 vs. 36% (3.34 [2.05–5.46]; *p* < 0.0001); more additional malignancies 81% versus 71% (*p* < 0.037, Yates correction); and family history of lung cancer in 2% versus 0.11% in controls <0.02, 16.69 [2.33–119.70] by Fisher’s exact test. The results are summarized in Table 1.

**Figure 2:**
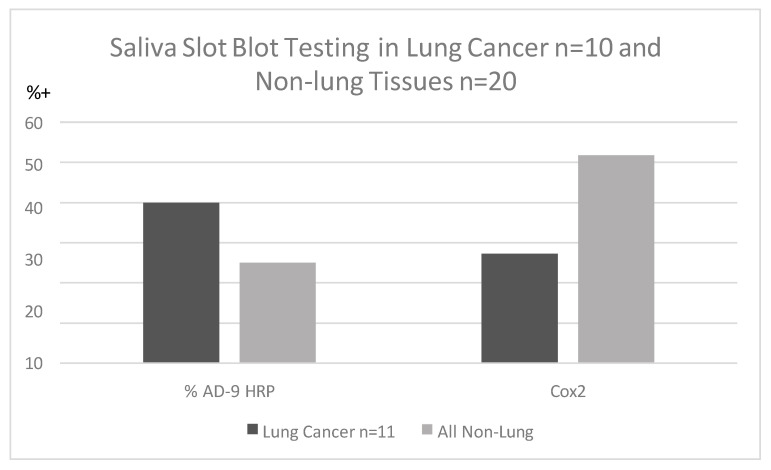
Percentage of Adnab-9 and anti-Cox2 binding to saliva (non-ordinal).

Saliva specimens collected before lung cancer diagnosis were reacted with p87 and OSN [9] for innate immune and adoptive immune systems but were not found to be clinically useful when standardized by protein contrast. Saliva peroxidase activity difference between patients and controls was significantly different despite relatively small numbers. Inherent salivary peroxidase activity in the LC group was significantly lower (OD < 0.05/1 µg protein) (*p* < 0.05) than in controls (64.3% positive). Although not significantly different, we found that Ad-9 bound to the saliva of lung cancer patients more than non-lung controls, and Cox2 antibody binding using horse-radish peroxidase to detect substrate showed an inverse relationship (Figure 2).

Figure 2 shows that saliva binding of Adnab-9 in slot blots is more sensitive in patients with lung cancer than Cox-2 antibody binding but inferior to historic stool and effluent binding, as noted above.

**Figure 3:**
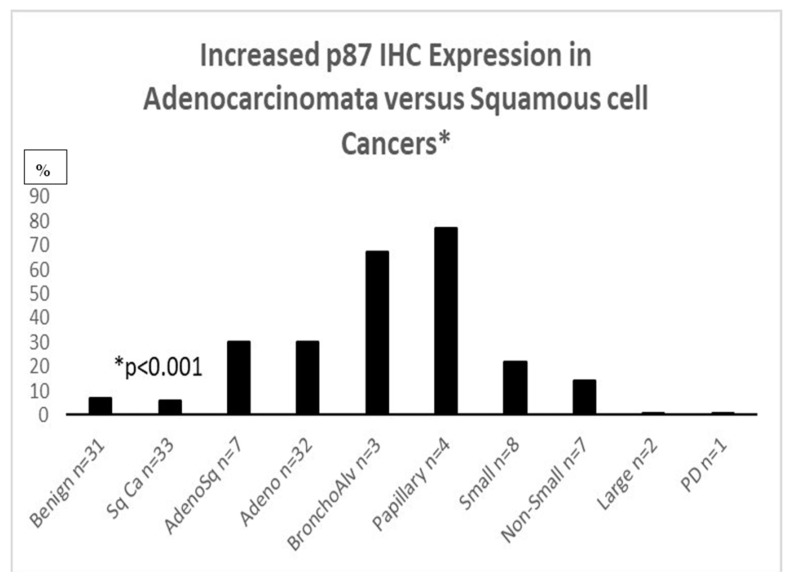
Adnab-9 IHC labeling of lung cancer slides.

**Figure 4:**
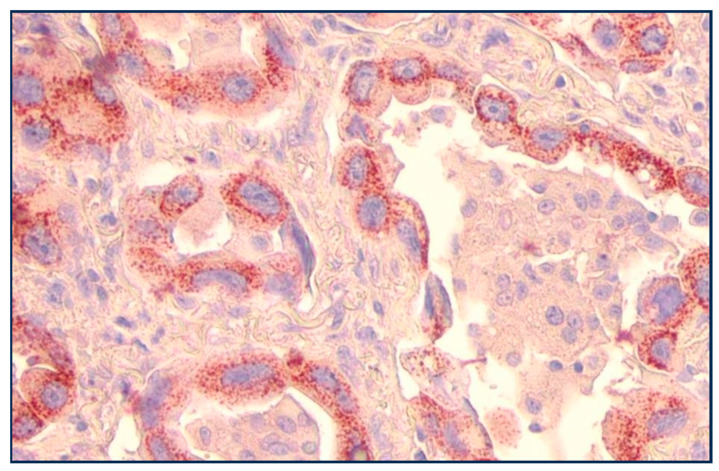
Bronchoalveolar lepidic-spread lung carcinoma with perinuclear p87 IHC (×20 magnification).

IHC for p87 favors certain phenotypes (Figure 3) and also shares an inverse relation with prognosis in the higher-graded tumors (Figure 4). Of seven lung cancer IHC samples, three were positive, and four were negative. Only one out of twenty-seven normal lung samples was positive (OR11.6 [CI1.04–128.97; *p* < 0.049]); see Figure 3 for a summary of the entire IHC group and individual cancers. All of the lung cancer KCI patients had a family history of lung cancer.

Figure 3: Bar diagram showing the percentage of various lung cancer phenotypes labeled with anti-p87 and Adnab-9. Bronchoalveolar (lepidic-spread) and papillary cancers were positive for labeling in over 60% (non-ordinal data).

**Figure 5:**
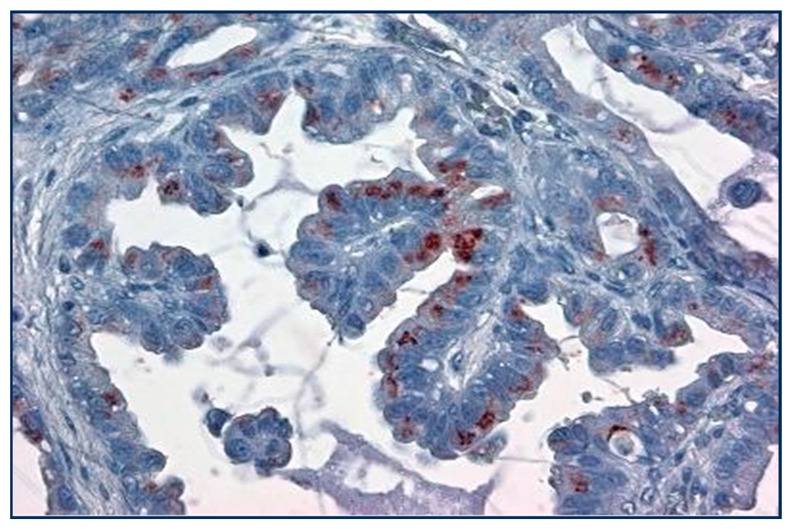
Papillary lung cancer with 1+ Golgi labeling with Adnab-9 monoclonal antibody (×20 magnification).

**Figure 6:**
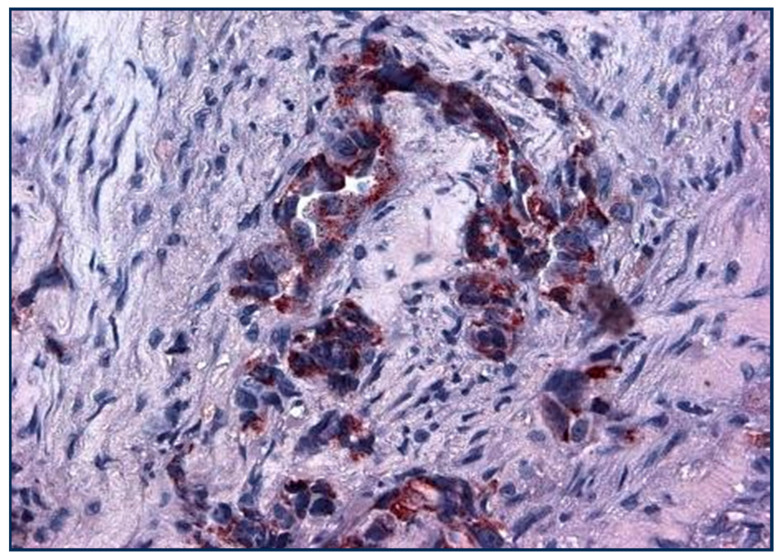
3 + Golgi staining of lung cancer cells with Adnab-9 (×20 magnification).

**Figure 7:**
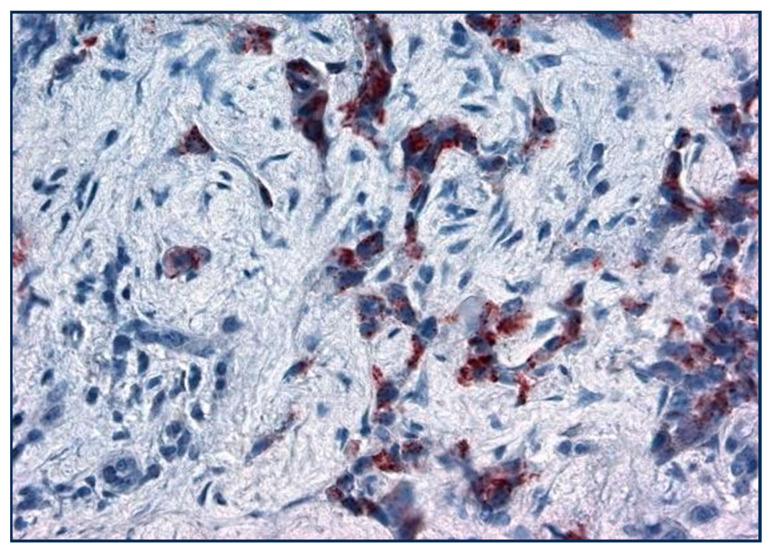
Lung cancer cells stained with Adnab-9 (×20 magnification).

**Figure 8:**
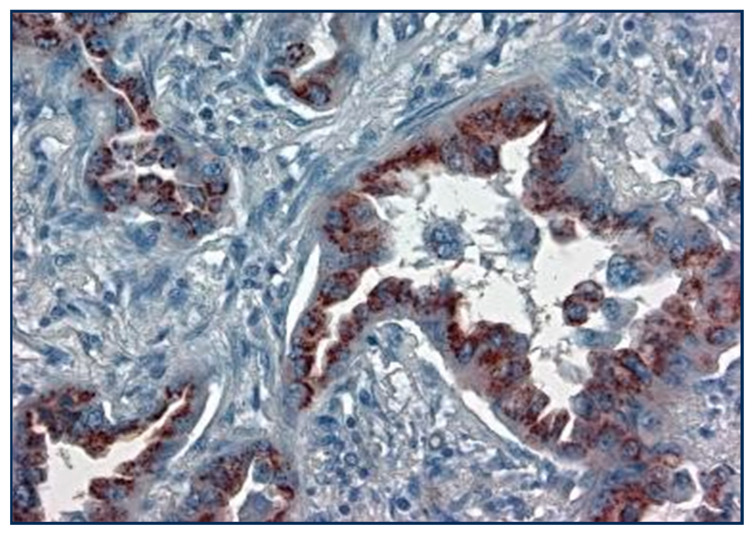
Golgi staining of p87 in adenocarcinoma lung cancer cells (×20 magnification).

**Figure 9:**
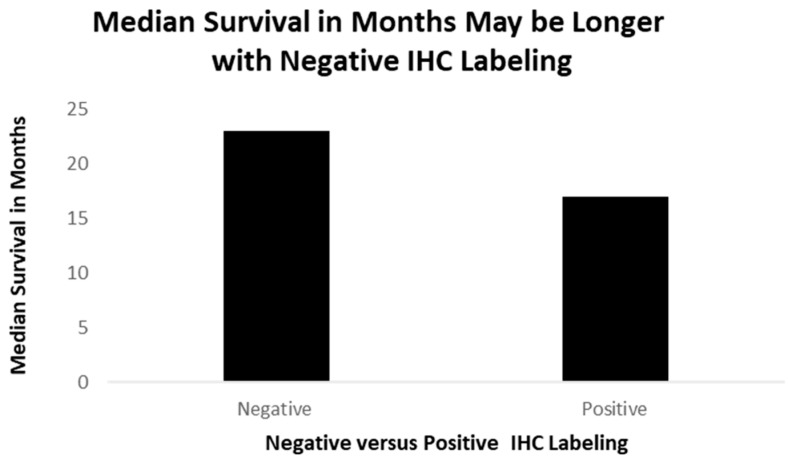
A better survival for patients (though not significant), where the lung cancer tissues stain negatively (non-ordinal data).

Figure 4, Figure 5, Figure 6, Figure 7 and Figure 8 are photomicrographs showing Adnab-9 labeling of representative sections of various forms of lung cancers. Figure 9 depicts survival in patients with positive IHC labeling versus those without.

Figure 4 shows a view of lung adenocarcinoma with Golgi staining and a lepidic form of infiltration. The substrate is ethylaminocarbazine (EAC), which, when present, is reddish-brown in color.

Papillary lung cancer at high power is seen to stain the Golgi apparatus with a reddish brown substrate but not uniformly.

Dense, focal, dark reddish p87 cytoplasmic deposits are seen in this invasive focus of NSCLC with a fibroblastic response.

The figure shows Golgi staining in adenocarcinoma with a less fibrocystic response.

Figure 8 shows that while the NSCLC cancer cells are positive, most of the non-cancer tissues do not take up the antibody.

The positive cases had, on average, 8.3 months of survival (one case is still alive and un-staged now, one had local disease, and one had distant disease), while the negative cases averaged 23 months of survival, depicted in Figure 8 (two with regional disease and two with distant disease).

Figure 9: A bar diagram showing survival times of 51 lung cancer patients, formalin-fixed and paraffin-embedded slides, staining for p87 (positive) and those without (negative).

When comparing primary lung cancer patients who had negative stool testing (*n* = 25) to those with positive testing (*n* = 26), there were no differences in demographics or survival (*p* = 0.35). Significant differences were seen in historic ferritin levels (358 ± 314 versus 149 ± 129; *p* < 0.034). Higher levels of native antigens in extracts of colonoscopy biopsies were found in the cecum and ascending colon in patients with negative p87 stool tests (cecum 0.132 ± 0.014 versus 0.055 ± 0.031; *p* < 0.035 and ascending colon 0.203 ± 0.059 versus 0.042 ± 0.023; *p* < 0.008), suggesting a lack of shedding from the right side of the colon that harbors Paneth cells reflected in the stool findings. The ratio of historic blood ferritin/fecal p87 (FERAD ratio), thought to reflect the activity of the InImS, was found to be commensurately high in patients with very low fecal p87 and higher ferritin levels (179,882 ± 302,992 versus 2987 ± 3492; *p* < 0.048). The patients with a negative stool and higher FERAD levels tended to survive longer than those with positive p87 stools but lower FERAD indices (10.6 ± 4.8 versus 7.3 ± 4.4 years, *p* = 0.35). The results are summarized in Table 2.

The interrelationship between p87 staining and lead-in time is quite complex and appears to be biphasic (Figure 8). The two axes intersect with the coordinates for squamous cell cancer at the intersection (oval symbol). There is a strong trend of a direct correlation between adenocarcinoma (faceted square) and large cell carcinoma (triangle) and a highly significant inverse correlation with NSLC at the upper end (pentagram) and small cell cancer (rectangle) at the lower end (Table 3). This suggests that for some p87 + cancers, prognosis is better, and for others, it is worse. This is not unusual for lung cancers [10], where morphologic characteristics are of profound importance for the type of treatment and, hence, prognosis. Certain assumptions were made that the p87% stain was uniform for the type of cancer since the data were derived from two different populations of patients.

**Figure 10:**
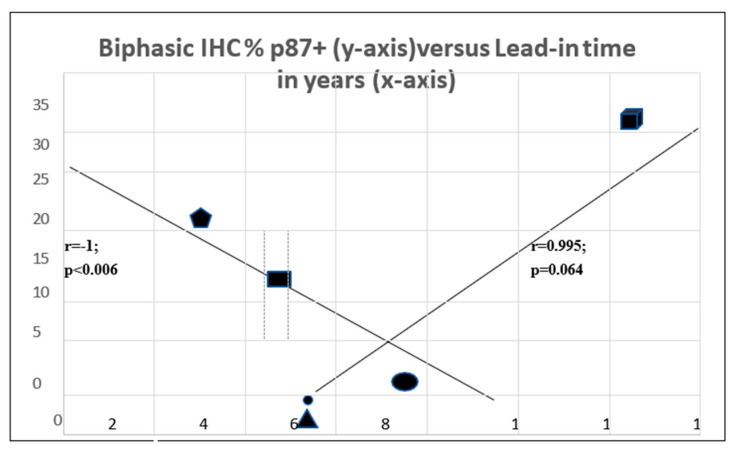
Complex relationship of lead-in time and % p87 staining.

Figure 10 symbols show a solid block square for adenocarcinoma, 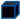; solid rectangle for NSCLC, 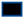; a solid circle for squamous cell, 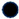; a solid triangle for large cell, 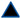; a solid pentagram for small >cell, 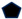.

**Figure 11:**
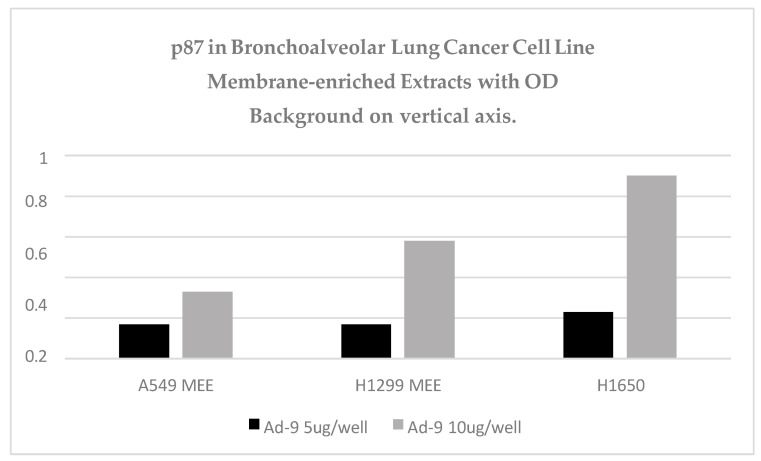
Three lepidic-spread lung adenocarcinoma cell lines were shown to be ELISA-positive.

Figure 11 shows the increase in binding with the increase in protein content in three lepidic-spread lung adenocarcinoma cell lines. H1650 p87 binding is equivalent to the positive control.

Adnab-9-treated lepidic bronchoalveolar cancer cell lines are antiproliferative, further supporting our observations.

**Figure 12:**
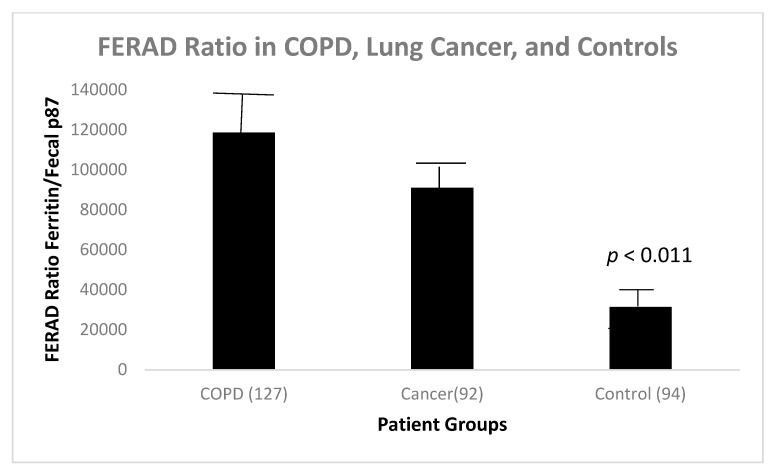
Bar diagram that depicts the FERAD ratio in disease states and controls.

We found that there was a statistically significant difference between the FERAD ratio in patients with COPD and controls (Figure 12).

**Figure 13:**
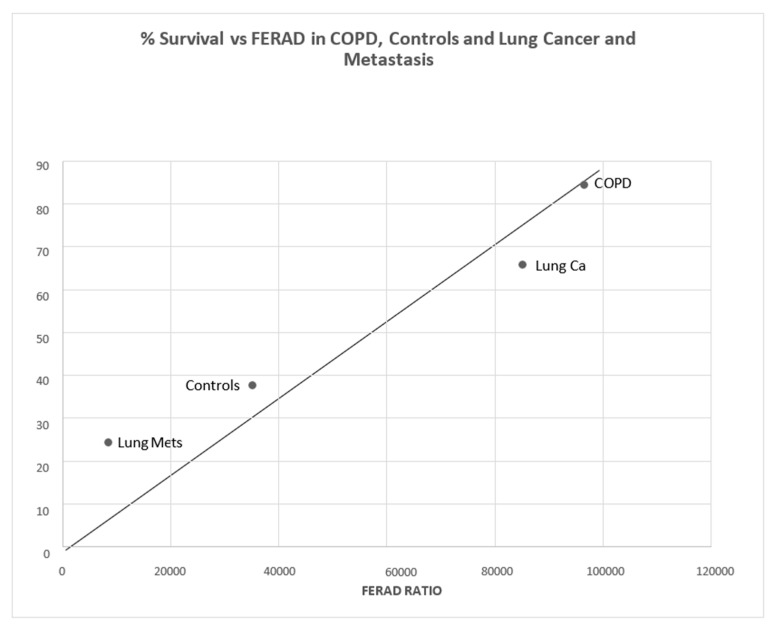
A positive correlation curve of survival versus FERAD ratio is depicted.

There is a statistical difference between COPD patients and controls (*p* < 0.011) in Figure 12. Interestingly, there is also a statistically significant positive correlation between survival in lung cancer patients, both primary and metastatic, and those with COPD and controls (Figure 13).

From left to right, the dots designate cancer metastatic (Mets) to lungs, primary lung cancer, COPD patients, and controls.

## 3. Discussion

p87 can detect cancers up to 12 years before they are diagnosed with reasonable sensitivity and specificity for the most common forms of lung cancer. This strategy can select outpatients at high risk who could undergo radiologic imaging, leading to a much earlier diagnosis that could currently be achieved. The p87 biomarker appears to identify patients with a poorer prognosis; thus, aiding in the detection of these tumors at a very early stage will allow for effective intervention. Hopefully, this approach will reduce mortality from this common organ cancer site. p87 staining suggests a differential stain in lung cancer as opposed to normal tissues. Conceptually, Adnab-9 recognizes the p87 antigen [8], a constituent of putatively activated pulmonary type 2 pneumocytes and, particularly, lung cancer cells. The p87 antigen is likely shed into sputum and swallowed, which may represent a useful potential tumor marker detectable by the Adnab-9 stool test. Of note is the fact that both α- Defensin5 and p87 are both Paneth cell secretory products, yet the former is not expressed in the lung. However, β-defensins are secreted in the lung, and earlier studies found a cancer association of overexpression in about 40–50% of common forms of lung cancer but no association of human β-defensins (HBD) type 1.2 and 4 with mRNA levels with histological type, tumor grade or stage of disease, or human levels of the peptide [12]. Earlier, others [13] had shown a correlation of HBD1 and 2 with lung cancer, with an estimated sensitivity of 76.4% and specificity of 94% pre-surgery but not prospectively with respect to cancer diagnosis that was known at the time of venesection. A later summary of the data for HBD1, 2, and 3 cautioned against generalization with different cancer types but allowed the association with tumor progression [14]. The same caveats may be applied to p87, but the lead-in time for diagnosis is, for now, unparalleled. Despite a similar cellular source as defensins, p87 outside of the lung does not usually label cancers but has been shown to be retrospectively diagnostic and prognostic in stool in both colorectal cancer (CRC), IPMN pancreatic subtype, and gastric cancer [3,15,16,17,18] and prospective in the common form of pancreatic cancer [19], where the lead-in time was 2.3 years. This and other works conform to biomarker standards proposed in 2018 [11], such as objective measuring to which our standardized ELISA aligns, evaluated as a usual biological process (product of the innate immune system), influence clinical decisions and minimize harm (p87 estimation is non-invasive, risk-free, and predictive); the biomarker must be stable and reproducible (p87 is stable for more than 10 years [19] in cold storage with a high correlation coefficient); must be sensitive and specific (while p87 is only moderately sensitive and specific ~60% for both, its lead-in time more than compensates, but the algorithm for use has, thus far, not been developed definitively). Adnab-9 has been confirmed to be prognostic by an independent laboratory [20], does not disrupt workflow, and reduces cost and harm. p87 is an inexpensive, cost-effective ELISA-based test, and envisaged follow-up would be down-to-top to minimize costs by allying the stool test with salivary peroxidase to increase specificity, and both could be sent in the mail on fecal occult blood cards, which has been shown to be acceptable and effective in large-scale [10] CRC screening trials. The medium should be convenient, such as lung sources (exhaled breath or sputum), but blood [21], saliva, and urine are also convenient (p87 could be co-opted onto the organ-specific neoantigen (OSN) urine test [9], which we also used in this study in limited numbers. Low-dose computerized tomographic (LDCT) screening has been shown to reduce disease-specific mortality from lung cancer by 20% and all-cause by 7%, but this has come at the cost of poor specificity, with one-quarter of screened patients exhibiting lesions and 96% proving to be false-positive associated with many drawbacks [22]. They conclude that new modalities are promising but do not satisfy current goals to qualify as biomarkers of proven utility.

Currently, the diagnosis of lung cancer utilizes different types of imaging complemented with biopsy assessment; however, these techniques can still not detect early lung cancer developments [23]. Other approaches, including the use of microRNA expression as genetic markers for lung cancer diagnosis, have been explored [24,25,26]. Immunohistochemistry (IHC) is being used as a rapid and cost-effective tool for the screening and detection of many of these markers [27].

There are many pitfalls, even with morphology used, in characterizing the type of lung cancer; for example, lepidic growth patterns, a more unusual characteristic of invasive mucinous lung adenocarcinoma (Figure 12), can be mistaken for metastatic pancreatic cancer [28,29]. Molecular testing, sometimes yielding similar results for both types of cancer, may confound the differential diagnosis [30]. Table 1 shows interesting differences; some can be explained by lifestyle differences, such as smoking and the effects of being overweight, but others are more challenging. Fewer “other cancers” are somewhat counter-intuitive. Black ethnicity is significantly more prevalent, but this may be a normal inner-city distribution. The lethality of lung cancer, despite significant advances, is still imposing. Family history differences may suggest familial commonality of risk factors.

While Table 2 shows no increased mortality based on p87 expression, the differences may be explained by increased shedding, particularly from the right colon where the colonic Paneth cells are distributed. If the numbers could be increased, the current trend of increased mortality in p87 + patients may become significant. The innate immune system, as defined by the FERAD ratio, suggests a significant reduction in p87 + patients, which may have associated adverse events. The H1650 cell line has an EGFR mutation in exon 19 but is very resistant to EGFR-directed inhibitor treatment despite seeing responses in other cell lines showing the same mutation [31]. It does express p87, and by using anti-p87 Adnab-9 monoclonal, proliferation is reduced. This monoclonal should be considered in treatment trials of cancers with positive EGFR mutations yet showing anti-EGFR resistance.

## 4. Conclusions

The role of COPD has been established as a concomitant of lung cancer, and we show, in Figure 12, elevated FERAD ratios in both COPD and lung cancer patients, supporting this notion that InImS reactivity correlates with both diseases equally. In Figure 13, we also show a direct correlation of FERAD with survival. We thus can diagnose lung cancer earlier by fecal p87 shedding and may also be able to prognosticate by serially following the FERAD ratio as a biomarker.

In addition, with the advent of a new monoclonal (Dupilumab) that blocks the shared receptor for Il-4 and IL-13 used in COPD patients with type 2 inflammation, the inflammation driving both COPD and lung cancer may help prevent the disease [32]. Conceivable changes could be detected with the FERAD ratio and could help manage these diseases. These data support the notion that accessible, early, non-invasive testing combined with effective follow-up modality may be able to reduce mortality from lung cancer, the world’s most frequent cancer, with reduced adverse events as the desirable outcome.

## Figures and Tables

**Table 1:** Demographic table of lung cancer cases vs. controls.

Parameter	% Lung Cancer Cases	% Controls	Chi-Square: or (ci)	*p*-Value
Age > 50	77.7	61.7	2.16 (1.37–3.40)	<0.001
Male gender	92.0	88.8	1.44 (0.72–2.89)	0.38
Race black	66.7	52.2	1.83 (1.22–2.75)	<0.005
BMI > 28	29.3	51.3	0.39 (0.24–0.64)	<0.0002
FEV1 > 1.5 L/s	89.5	83.7	1.66 (0.37–7.53)	0.55
Smoking	65.3	36.1	3.34 (2.05–5.46)	<0.0001
Diabetes	26.1	36.2	Yates cs 3.25	0.07
No F/H cancer	45.3	41.2	Yates cs 0.53	0.47
No other cancer	18.9	28.5	Yates cs 4.34	0.037
Mortality	87.5	61.1	0.23 (0.13–0.40)	<0.0001
F/H Lung Ca	1.9	0.11	16.69 (2.33–119.70)	<0.02

or—odds ratio; ci—confidence intervals; >—greater than; <—less than; BMI—body mass index; FEV1—forced expiratory volume in 1 s; /—per; F/H—family history; cs—chi-square.

**Table 2:** Comparison of patients with stool p87 over the cutoff threshold (p87+) and below (p87−).

Parameter	p87− Patients *n* = 26	p87+ Patients *n* = 25	*p*-Value
Age in years ± standard deviation	63.2 ± 9.66	67.5 ± 11.2	*p* = 0.149
Survival (dx to death)	10.6 ± 4.8 years	7.3 ± 4.4 years	*p* = 0.35
Ferritin (ng/dL) ± standard deviation	358 ± 314	149 ± 129	*p* < 0.034
Native p87 cecum ELISA (OD-Bg)	0.132 ± 0.014	0.055 ± 0.031	*p* < 0.035
Native p87 Asc C ELISA (OD-Bg)	0.203 ± 0.059	0.042 ± 0.023	*p* < 0.008
FERAD ratio	179,882 ± 302,992	2987 ± 3492	*p* < 0.048

− = negative; +—positive; dx—diagnosis; Asc C—ascending colon; OD—optical density; Bg—background; FERAD—ferritin/fecal p87.

**Table 3:** Comparison of percentage labeling with other common lung cancer antigens, as previously described [11].

Cancer Type	Ad-9 (p87)	TTF1 (Clones)	NE Marker	%p63/p40	CK7 (NRecc)
Lepidic/BA	67		N/A	N/A	
AdenoCa	32	80 (muc10–15)	N/A	20–30	94–100
Squamous	6		N/A		5–77 mean 25%
Large Cell	0		41 (9–85)	92	
Small Cell	22		79–100		
NSCLC	15		10–20	68	
AdenoSquamous	27		N/A	99	
Papillary	74		N/A		

NE = neuroendocrine

## Data Availability

The data presented in this study are available in this article.
